# Supplementary material for: Rapid fabrication of gold microsphere arrays with stable deep-pressing anisotropic conductivity for advanced packaging
Source: Nat Commun. 2024 Oct 24;15:9182. doi: 10.1038/s41467-024-53407-x (PMC11502786; doi:10.1038/s41467-024-53407-x)
Supplement: Supplementary file 1 — Supplementary Information [file 41467_2024_53407_MOESM1_ESM.pdf]

Supplementary Information

Rapid Fabrication of Gold Microsphere Arrays with Stable Deep-pressing Anisotropic Conductivity for Advanced Packaging

An Cao[1,#], *Yi Gong*[1,2,#], *Dilong Liu*[1,#,*], *Fan Yang*[3], *Yulong Fan*[4,5]*, *Yinghui Guo*[4,5], *Xingyou Tian*[1], *and Yue Li* [1,3]*

1. Key Lab of Materials Physics, Anhui Key Lab of Nanomaterials and Nanotechnology, Institute of Solid State Physics, HFIPS, Chinese Academy of Sciences, Hefei 230031, P. R. China

2. China-Europe Electronic Materials International Innovation Center, Hefei 230000, P. R. China

3. Tiangong University, Tianjin 300387, P. R. China

4. National Key Laboratory of Optical Field Manipulation Science and Technology, Chinese Academy of Sciences, Chengdu 610209, P. R. China

5. State Key Laboratory of Optical Technologies on Nano-Fabrication and Micro-Engineering, Institute of Optics and Electronics, Chinese Academy of Sciences, Chengdu 610209, P. R. China

6. Quantum Science Center of Guangdong-HongKong-Macao Greater Bay Area (Guangdong), Shenzhen 518045, P. R. China

# These Authors contributed equally to this work.

*Address correspondence to dlliu@issp.ac.cn, yulong_fan2017@163.com, yueli@issp.ac.cn

This PDF file includes:

Supplementary Notes 1 to 2

Supplementary Figures 1 to 20

Supplementary Table 1

Supplementary References

Supplementary Note 1: Theoretical diameter decreases from gold colloidosomes into gold microspheres.

Since gold colloidosomes are assembled as a hexagonally close-packed model, the distance change of colloidosomes into microspheres is calculated using close packing model. The close-packed structure can be reduced to the ordered arrangement of an infinite number of identical cells, so we only need to study the space utilization of a single cell. As shown in the Supplementary Fig. 7a, the unite cell shape of the close-packed structure is hexagonal prism. The volume of the prism is the volume of the unite cell (equivalent to the volume of the colloidosome).

Assuming that the size of each gold particle is uniform and the radius is r, then the volume of a gold particle is $v_a = \frac{4}{3}\pi r^3$. Each gold particle on the 12 vertices of the unite cell is equally divided by six adjacent unite cells, and each unite cell is divided by 1/6, so the number of particles divided by each vertex of a crystal cell is one. So, the total number of particles divided by a cell is $12\times\frac{1}{6}=2$. Each gold particle on the upper bottom surface and the lower bottom surface is equally divided by two adjacent unite cells, so the number of gold particles divided by the upper bottom surface and the lower bottom surface of a cell is $2\times\frac{1}{2}=1$. The three gold particles in the middle are owned by this cell alone.

Therefore, this unite cell contains a total of six gold particles, and the total volume of gold particles (equivalent to the volume of gold microsphere) is $V_a=6\times\frac{4}{3}\pi r^3=8\pi r^3$.

The shape of the unite cell of gold colloidosome is prismatic, assuming the base area is S and the height is H. So, the volume of the cell is $V_b=SH$. The top view of the base is shown in Supplementary Fig. 7b, and the area is $S=6\sqrt{3}r^3$. As shown in

Supplementary Fig. 7c, the three gold particles of the second layer are represented by blue circles. The central gold particle of the bottom surface and the first gold particle of the second layer are observed. The center distance of the two gold particles is obviously $2r$, but the height difference Δh is unknown. The horizontal distance is $\frac{2\sqrt{3}}{3}r$ (according to the Pythagorean theorem). As shown in Supplementary Fig. 7d, the Δh between the bottom gold particle and the middle gold particle can be calculated according to the Pythagorean theorem, that is

$$\Delta h=\sqrt{(2r)^2-\left(\frac{2\sqrt{3}}{3}r\right)^2}=\frac{2\sqrt{6}}{3}r \quad (1)$$

$$H=2\Delta h=\frac{4\sqrt{6}}{3}r \quad (2)$$

$$V_b=S\times H=6\sqrt{3}r^2\times\frac{4\sqrt{6}}{3}r=24\sqrt{2}r^3 \quad (3)$$

The diameter conversion rate (σ) of gold colloidosomes to gold microspheres. It is assuming that the volume conversion rate of colloidosome into microsphere isηunder the close pecking model.

$$\eta=\frac{V_a}{V_b}=\frac{8\pi r^3}{24\sqrt{2}r^3}=0.74 \quad (4)$$

In order to facilitate experimental statistics, the volume conversion rate was ηconverted to direct conversion rateσ, therefore,

$$\sigma=\sqrt[3]{\eta}=\sqrt[3]{0.74} \quad (5)$$

Therefore, in the ideal close-packed case, $D_{sphere}=\sqrt[3]{0.74}D_{colloids}$.

Supplementary Note 2: Calculation of theoretical relationship between laser pulse fluence ($J(\tau)$) and diameter of gold colloidosomes (d_p).

On the basis of previous research reports on the heating-melting-fusion mechanism,[1] we have made some simplifications to the model to understand the relationship between the laser fluence and the diameter of gold colloidosome and give as follow.

A laser energy E_{abs} that gold colloidosome can absorb from a single laser pulse is equal to

$$E_{abs}=J\sigma_{abs}^{\lambda}(d_p) \tag{6}$$

In this equation, J is laser fluence, $\sigma_{abs}^{\lambda}(d_p)$ is the diameter d_p dependent absorption cross section of gold colloidosome, which is related to laser wavelength λ and refractive index of surrounding medium n_m. The laser energy E_{abs} absorbed by the gold colloidosome is used to heat the lattice, and when the temperature reaches the melting or boiling point of gold (1336 and 3150 K respectively), the gold colloidosome will melt or evaporate, as follow:[1]

$$E_{abs}(d_p)=\rho_p\left(\frac{\pi d_p^3}{6}\right)\left[\int_{T_0}^{T_m} C_p^s(T)\mathrm{dT}+\Delta H_m+\int_{T_m}^{T_b} C_p^l(T)\mathrm{dT}\right]$$

$$=\rho_p\left(\frac{\pi d_p^3}{6}\right)\left[(H_{T_m}-H_{T_0})+\Delta H_m+(H_{T_b}-H_{T_m})\right] \tag{7}$$

Here, $E_{abs}(d_p)$ is the diameter dependent energy required to melt the gold colloidosome, ρ_p is density of gold, T_0, T_m, T_b are room temperature, melting temperature and boiling temperature respectively, C_p^s and C_p^l are the gold nanoparticles heat capacities in solid and liquid states, ΔH_m is the enthalpy of melting, and H_T-H_{T_0} is the relative enthalpy. Considering that the evaporation phenomenon can be ignored in this experiment due to the small fluence (<50 mJ·cm^{-2}) and short time (<10 s), Equation (7) can be simplified as follow:

$$E_{abs}(d_p)=\rho_p\left(\frac{\pi d_p^3}{6}\right)\left[(H_{T_m}-H_{T_0})+\Delta H_m\right] \quad (8)$$

Integrated with the equation (6), the general relationship between the size of gold nanoparticles (d_p) and laser fluences (J) is given in equation (9):

$$J(d_p)\sigma_{abs}^{\lambda}(d_p)=\rho_p d_p\left(\frac{\pi d_p^3}{6}\right)\left[(H_{T_m}-H_{T_0})+\Delta H_m\right] \quad (9)$$

Where $J(d_p)$ is the diameter dependent laser fluence required to melt the gold colloidosomes. According to Fig. 3b, the $\sigma_{abs}^{\lambda}(d_p)$ can be expressed as:

$$\sigma_{abs}^{\lambda}(d_p)=K_{absorption}\times d_p-a \quad (10)$$

Where $K_{absorption}$=0.00905 and a=2.0725. Relate equation (10) to equation (9), we can have finally the dependence of required laser fluence on colloidosome's diameter:

$$J(d_p)=\rho_p\left(\frac{\pi}{6}\right)\left[(H_{T_m}-H_{T_0})+\Delta H_m\right]\frac{d_p^3}{K_{absorption}\times d_p-a}=C\frac{d_p^3}{K_{absorption}\times d_p-a} \quad (11)$$

Where $C=\rho_p\left(\frac{\pi}{6}\right)\left[(H_{T_m}-H_{T_0})+\Delta H_m\right]$ is a constant for gold. When d_p is larger than $\frac{a}{K_{absorption}}$, $J(d_p)$ follows d_p^2 lineshape. Take $K_{absorption}$=0.00905 and $a\approx2.07$ into equation (11), we plot the colloidosome's diameter dependent laser fluence for melting in Supplementary Fig. 13, which is in line with the experimental result revealed in Figure 2b when d_p>400 nm.

Therefore, given a gold colloidosome's size, there is a laser fluence threshold for the gold colloidosome that can be melted by one pulse. At the same time, because the quenching time is much smaller than the interval between the two pulses, the energies of the two lasers cannot be accumulated, so such a threshold cannot be broken even if the number of the pulse output is increased. This result is consistent with our experimental observation.

.

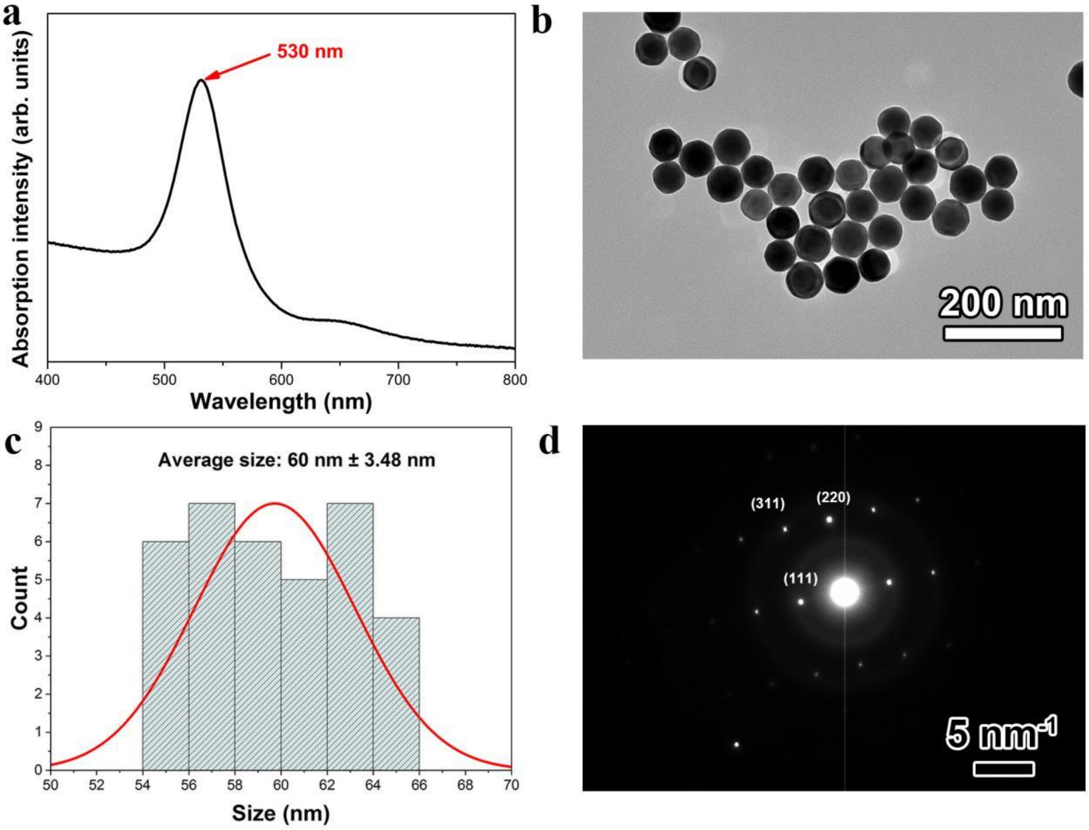

Supplementary Figure 1. Gold nanoparticles synthesized by polyol reduction strategy. a Absorption spectrum of gold nanosphere. **b** TEM image of raw gold nanoparticles. **c** Histogram of size statistics of the raw gold nanoparticles. **d** SAED pattern of raw gold nanosphere. Source data are provided as a Source Data file.

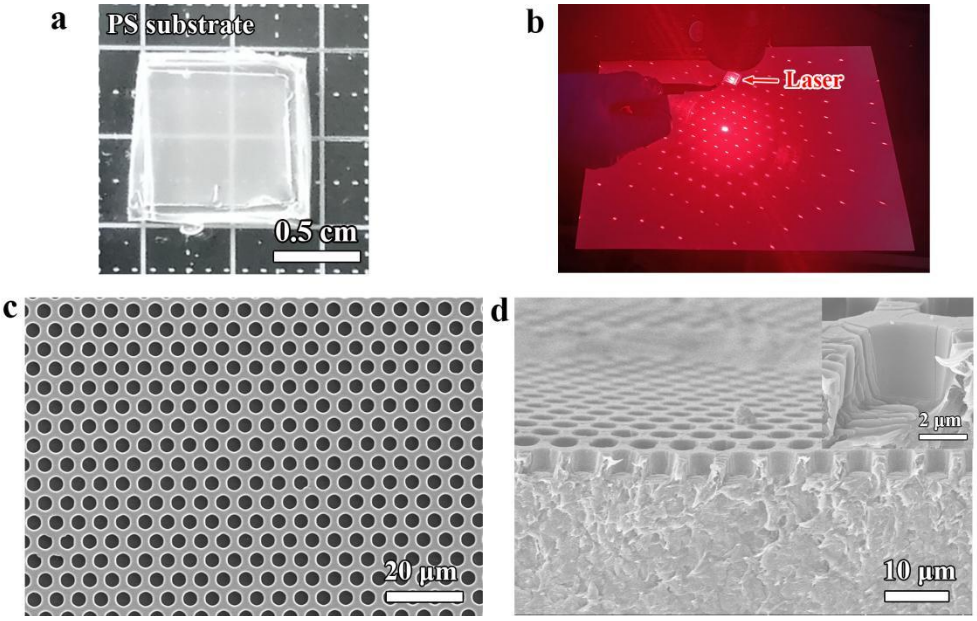

Supplementary Figure 2. The honeycomb PS substrate. a Digital photograph of honeycomb PS substrate. **b** Diffraction pattern of the 2D honeycomb PS substrate irradiated by a 633 nm laser pen. **c** SEM image of honeycomb PS substrate. **d** SEM image of honeycomb PS substrate at a cross-sectional view, and the inset showing the high-magnification SEM image of microhole.

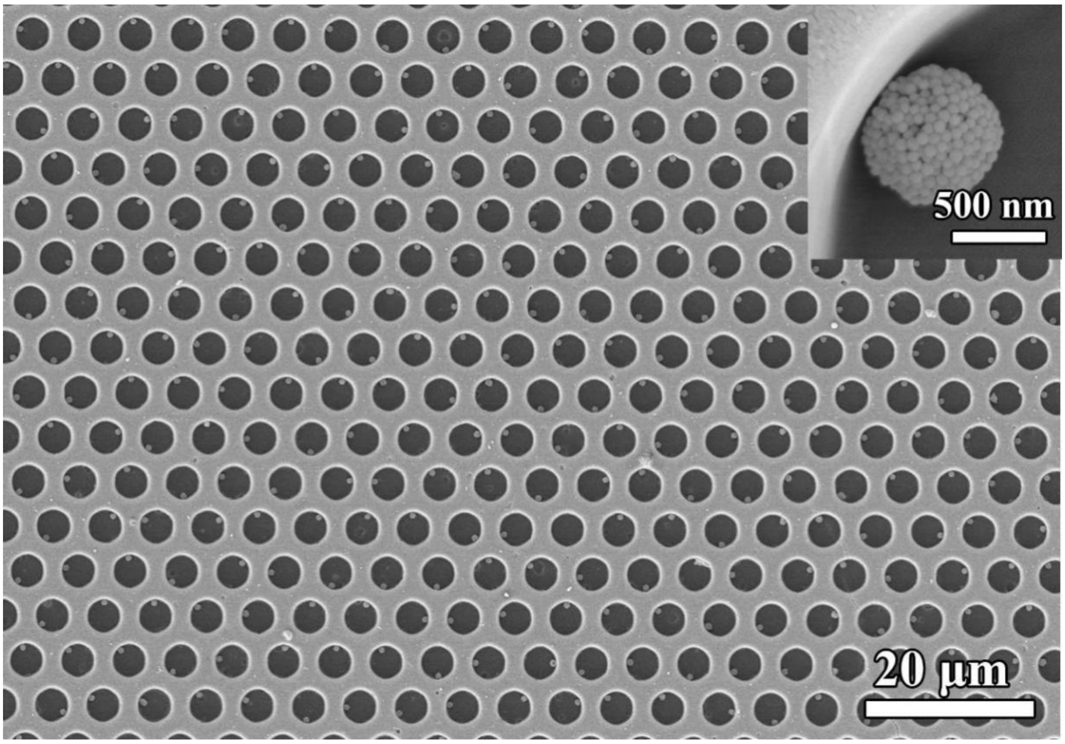

Supplementary Figure 3. Typical SEM image of gold colloidosomes array. Inset showing the high-magnification SEM image of single gold colloidosome.

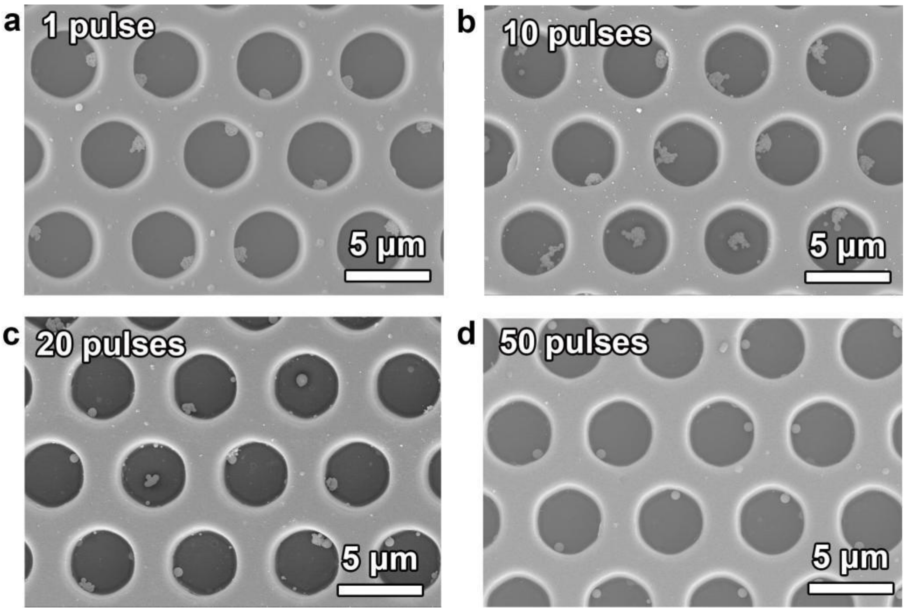

Supplementary Figure 4. SEM images of gold colloidosomes treated with different laser pulses. (a-d) The number of laser pulses are 1, 10, 20, 50 respectively.

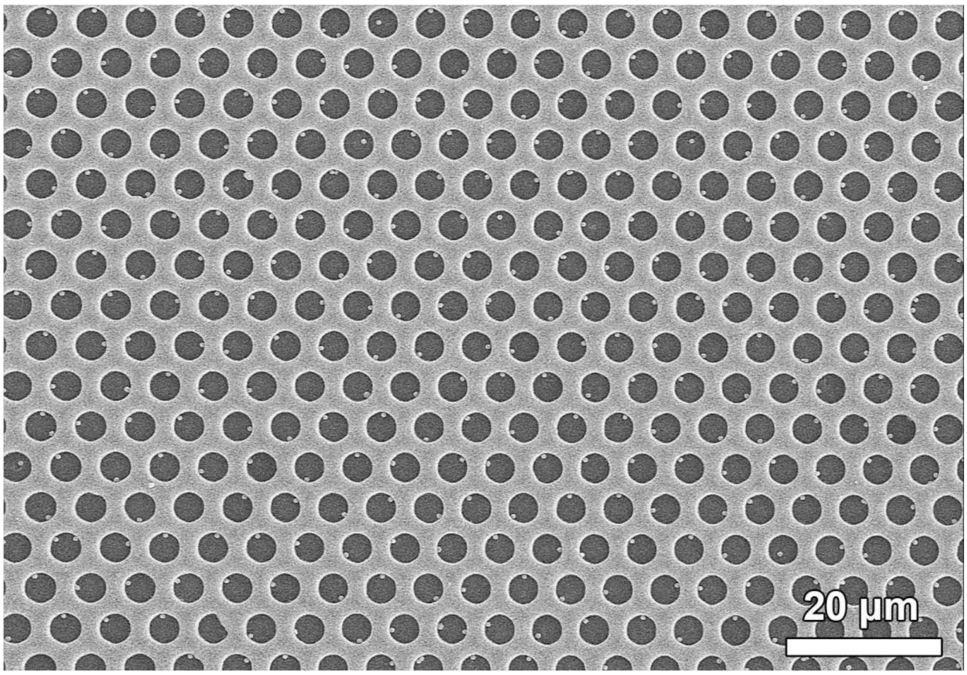

Supplementary Figure 5. Low-magnification SEM image of gold microspheres prepared based on layer-by-layer melting mechanism.

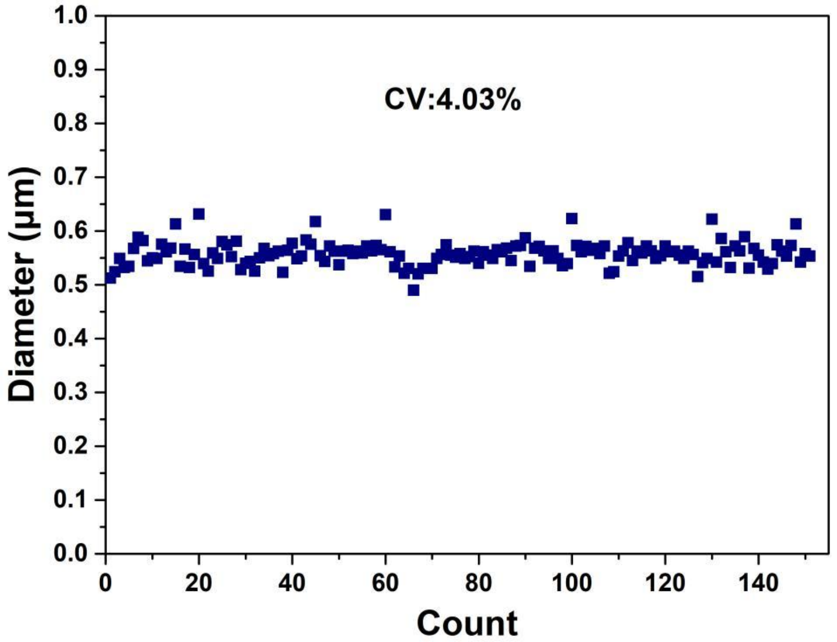

Supplementary Figure 6. The diameter of gold microspheres exhibits a coefficient of variation (CV) of 4.03%. Source data are provided as a Source Data file.

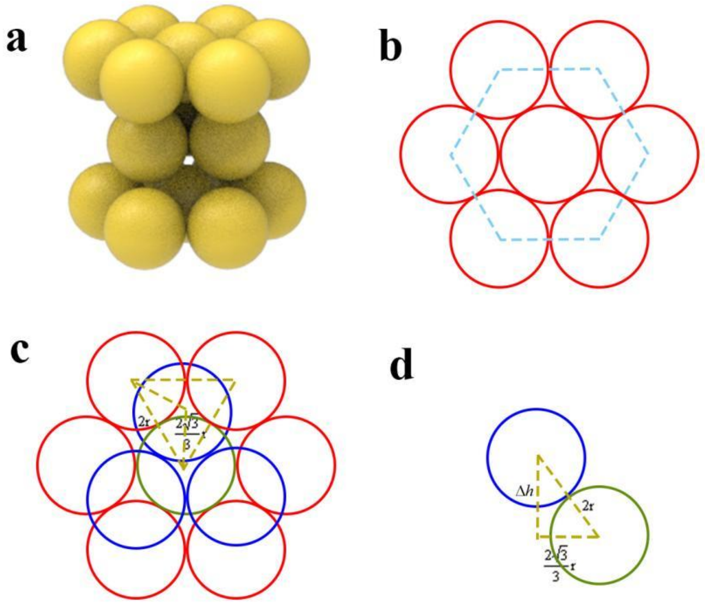

Supplementary Figure 7. The close packing model. a Schematic diagram of unite cell of the close packing model. **b** Top view of six bottom gold particles in a close packing model. **c** Top view of six bottom gold particles and three interlayer gold particles. **d** Side view of bottom intermediate gold particle and upper gold particle.

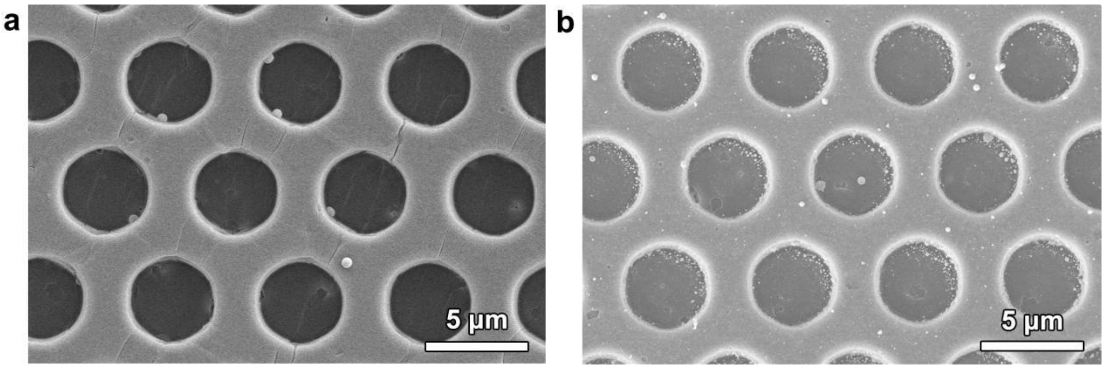

Supplementary Figure 8. The typical SEM images of gold microspheres when the laser fluence was excessive. a SEM image of gold microspheres after irradiated when laser fluence was 30.0 mJ·cm^{-2}. **b** SEM image of gold microspheres after irradiated when laser fluence over 50.0 mJ·cm^{-2}.

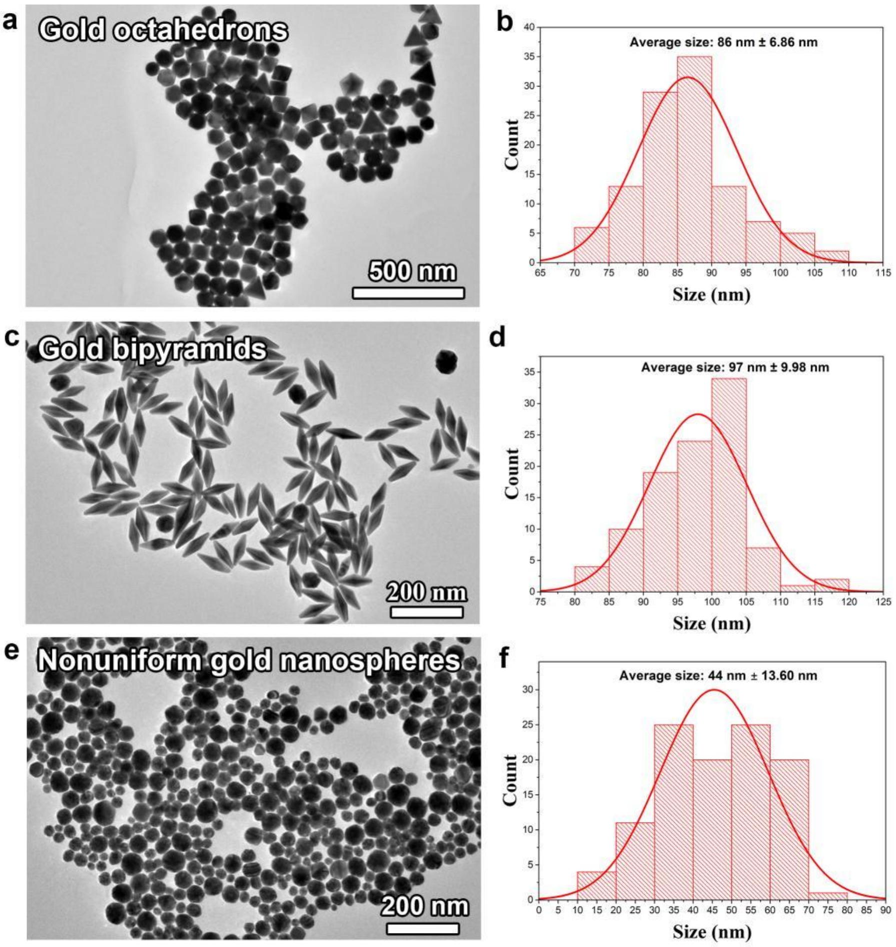

Supplementary Figure 9. Gold nanoparticles with different morphologies. a TEM image of gold octahedrons. **b** Histogram of size statistics of gold octahedrons. **c** TEM image of gold bipyramids. **d** Histogram of size statistics of gold bipyramids. **e** TEM image of nonuniform gold nanoparticles. **f** Histogram of size statistics of nonuniform gold nanoparticles. Source data are provided as a Source Data file.

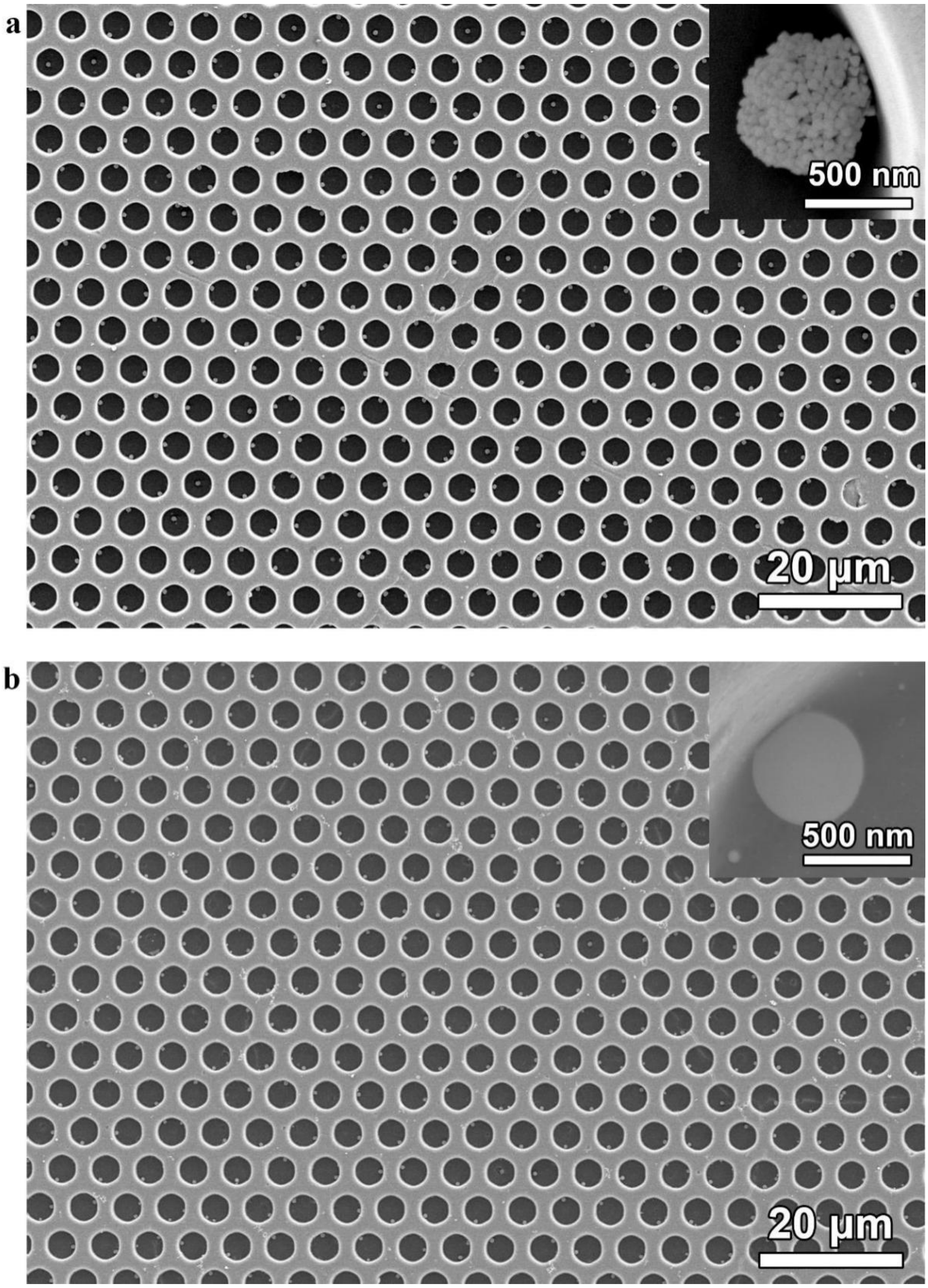

Supplementary Figure 10. Typical SEM images of gold colloidosomes and gold microspheres when building block was gold octahedrons. a Low-magnification SEM image of the gold colloidosomes with gold octahedrons, and inset showing the high-magnification SEM image of the gold colloidosomes. **b** Low-magnification SEM image of the gold microspheres and inset showing the high-magnification SEM image of the gold microsphere.

a

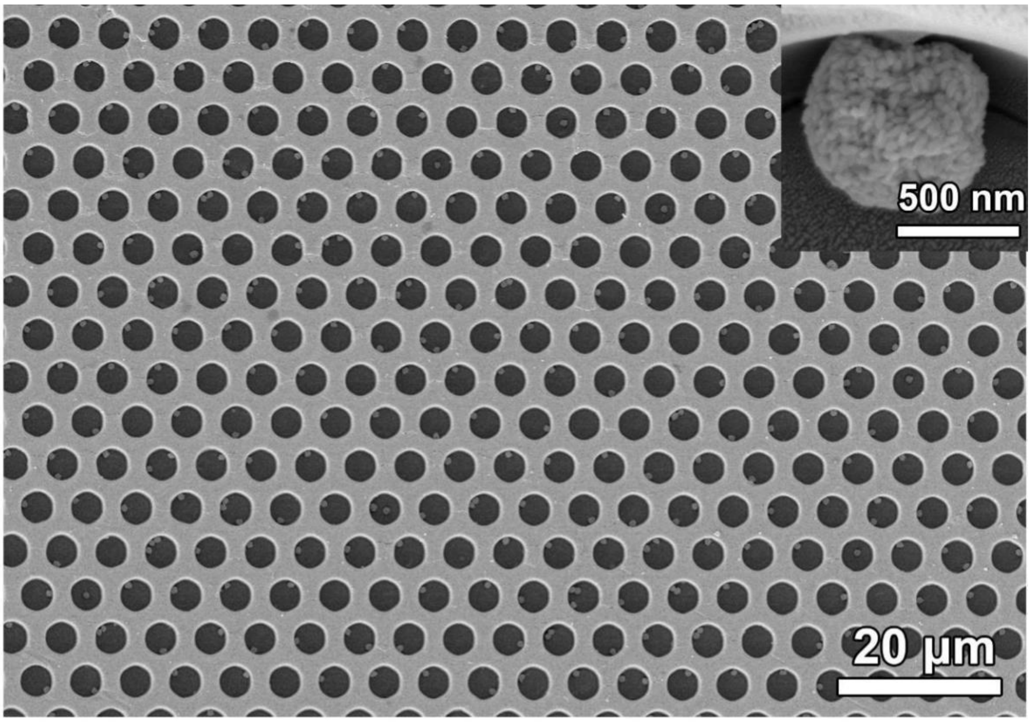

b

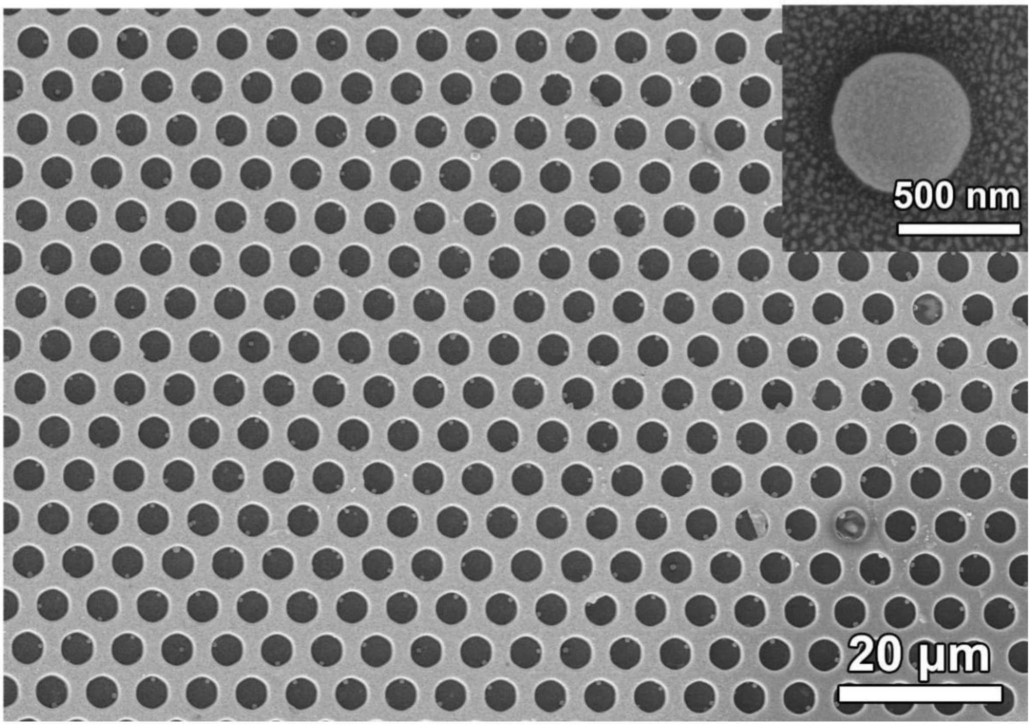

Supplementary Figure 11. Typical SEM images of gold colloidosomes and gold microspheres when building block was gold bipyramids. a Low-magnification SEM image of the gold colloidosomes with gold bipyramids, and inset showing the high-magnification SEM image of the gold colloidosomes. **b** Low-magnification SEM image of the gold microspheres and inset showing the high-magnification SEM image of the gold microsphere.

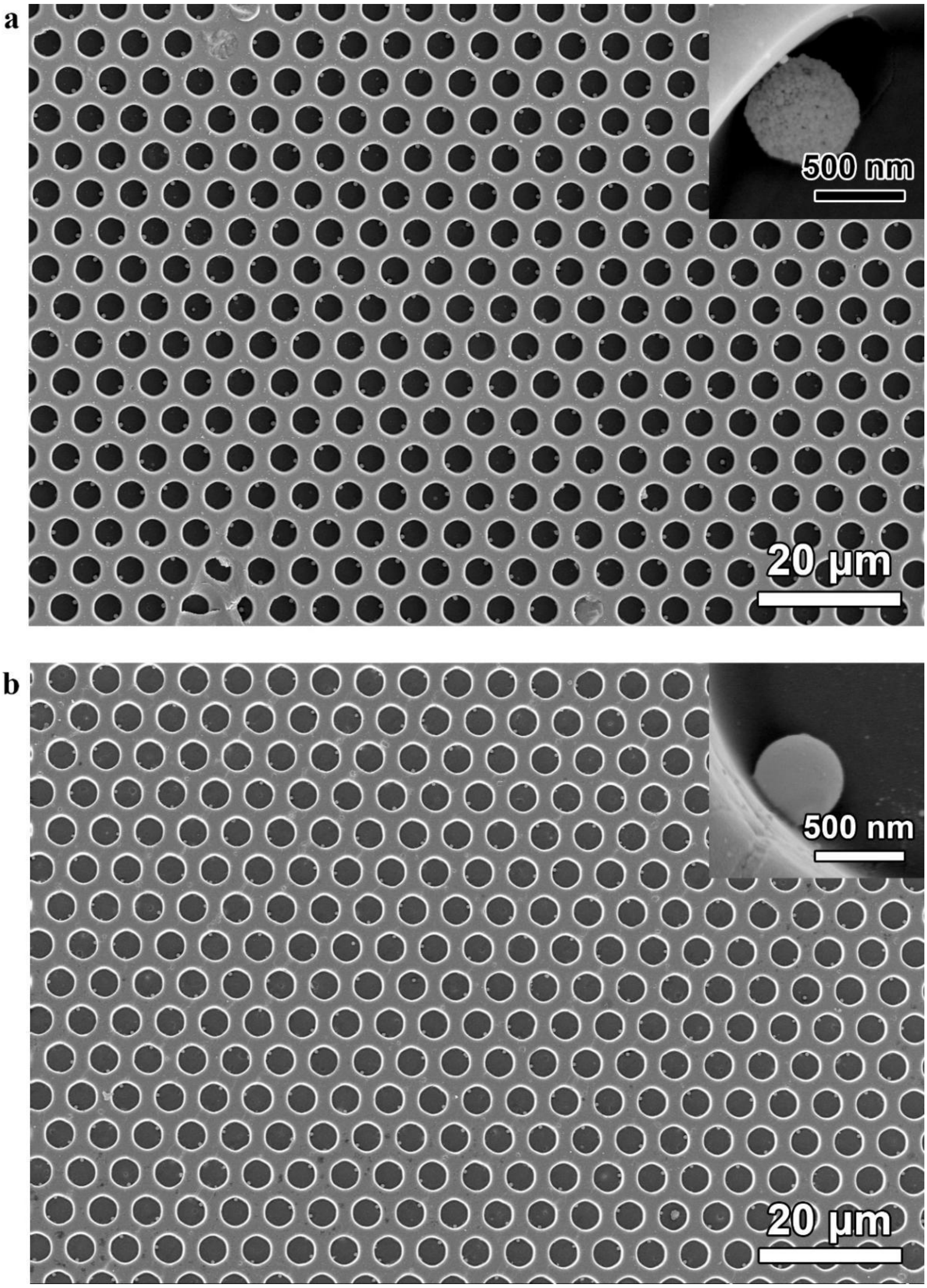

Supplementary Figure 12. Typical SEM images of gold colloidosomes and gold microspheres when building block was nonuniform gold nanoparticles. a Low-magnification SEM image of the gold colloidosomes with nonuniform gold nanoparticles, and inset showing the high-magnification SEM image of the gold colloidosomes. **b** Low-magnification SEM image of the gold microspheres and inset showing the high-magnification SEM image of the gold microsphere.

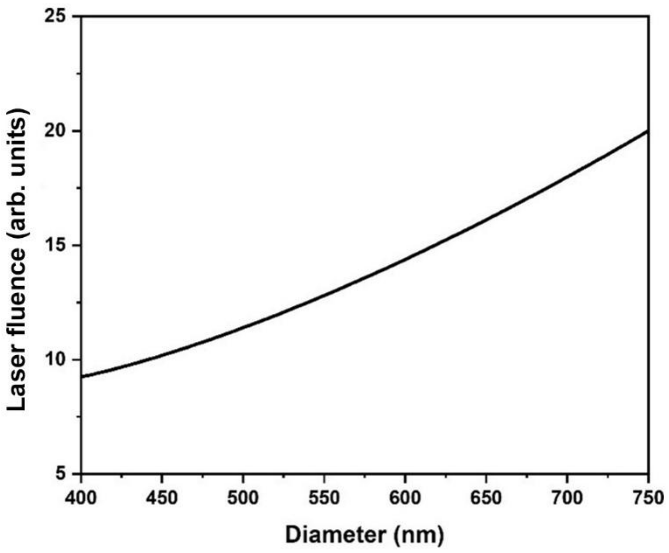

Supplementary Figure 13. **Diameter dependent laser fluence required to melt gold colloidosomes according to equation (11).** Source data are provided as a Source Data file.

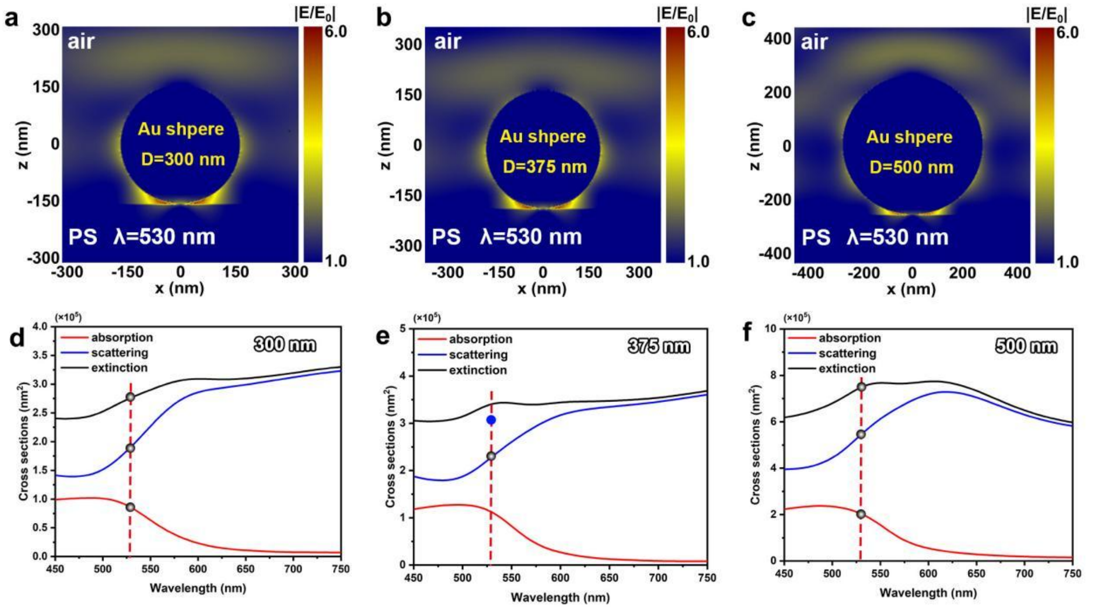

Supplementary Figure 14. **Simulated electromagnetic field distribution of gold microspheres. (a-c)** The simulated 2D electromagnetic filed distribution of gold microspheres. **(d-f)** The calculated cross sections of absorption, scattering and extinction of gold microspheres (the diameter of gold microsphere is 300, 375 and 500 nm). Source data are provided as a Source Data file.

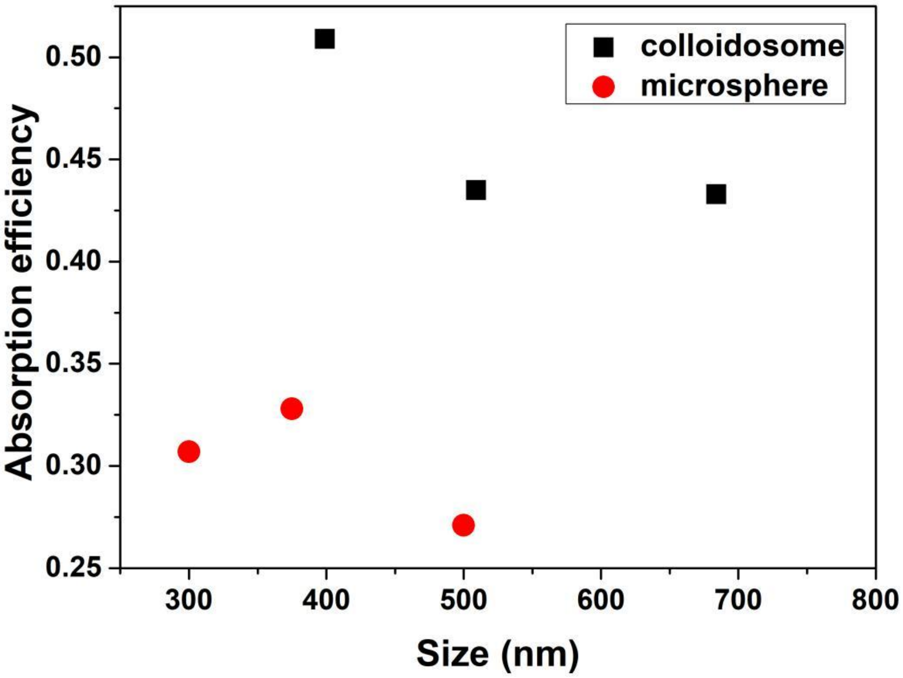

Supplementary Figure 15. **Absorption efficiency of gold microspheres and gold colloidosomes.** The black squares represent gold colloidosomes and the red circles represent gold microspheres. Source data are provided as a Source Data file.

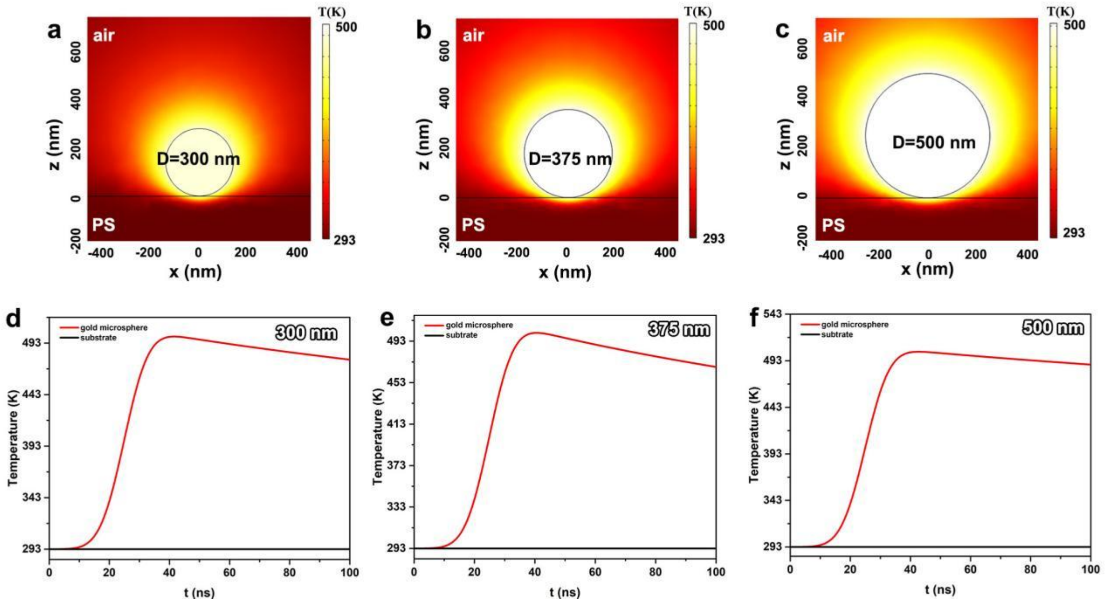

Supplementary Figure 16. **Simulated temperature evolution of the gold microspheres over time during laser pulse irradiation. (a-c)** Thermal distribution of the microsphere when gold microsphere achieves the most elevated temperature. **(d-f)** Temperature evolution curves of gold microspheres. (the diameter of gold microsphere is 300, 375 and 500 nm) Source data are provided as a Source Data file.

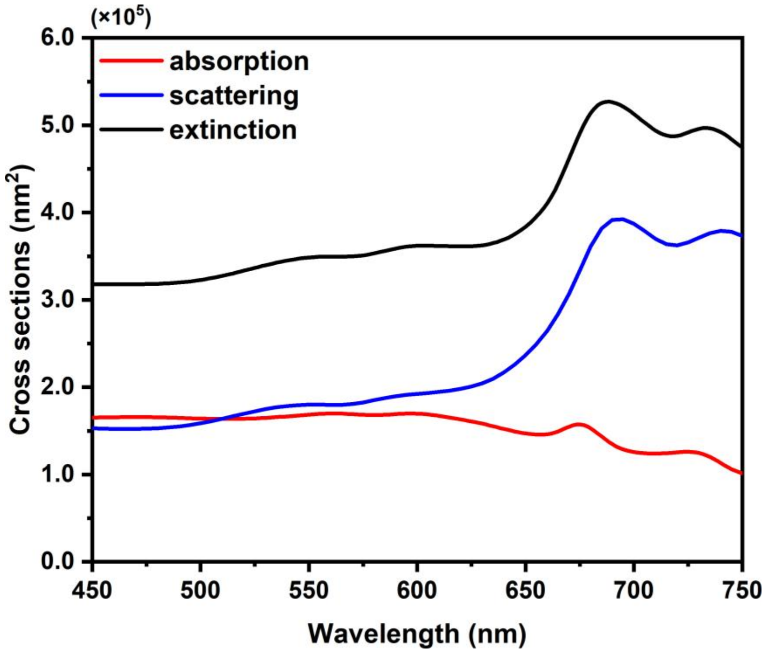

Supplementary Figure 17. **The calculated cross sections of 399 nm gold colloidosomes when environmental medium is 1-butanol.** The red line, blue line and black line represent the cross section of absorption, scattering and extinction respectively. Source data are provided as a Source Data file.

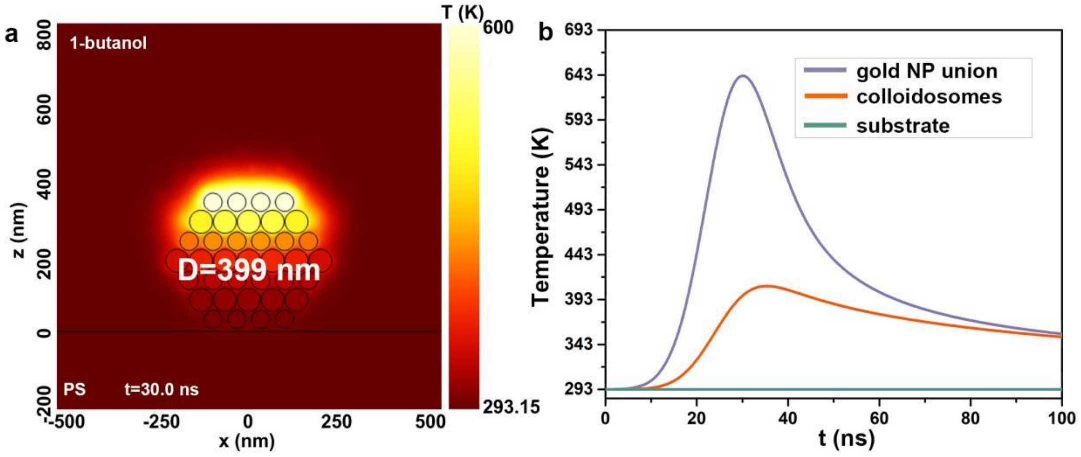

Supplementary Figure 18. **Simulated temperature evolution of the 399 nm gold colloidosomes over time during laser pulse irradiation when the environmental medium is 1-butanol. a** Thermal distribution of the colloidosome when gold colloidosomes achieve the most elevated temperature. **b** Temperature evolution curves of gold colloidosomes. The purple line, orange line and green line represent the temperature evolution of gold NP union, colloidosomes and substrate. Source data are provided as a Source Data file.

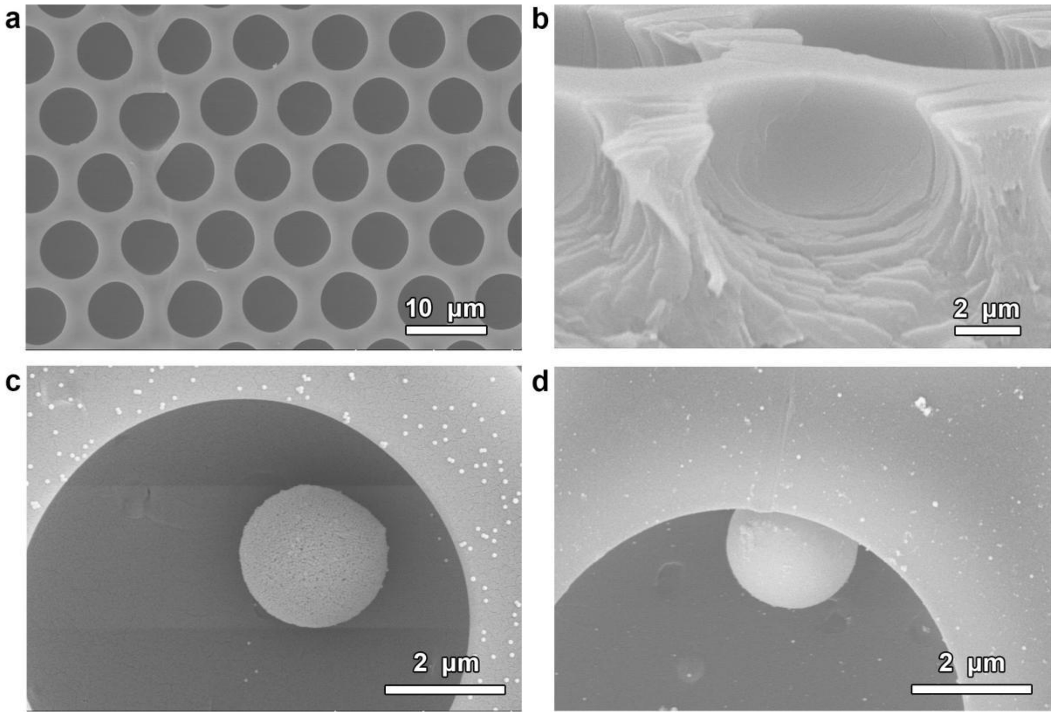

Supplementary Figure 19. **Preparation of 2 μm gold microspheres using larger templates. a** Typical SEM image of the PS substrate with large-sized microholes. **B** Cross-sectional SEM image of (**a**). **c** Typical SEM image of 2.5 μm gold colloidosomes obtained by the transient emulsion self-assembly. **d** Typical SEM image of 2.0 μm gold microspheres after the laser irradiation.

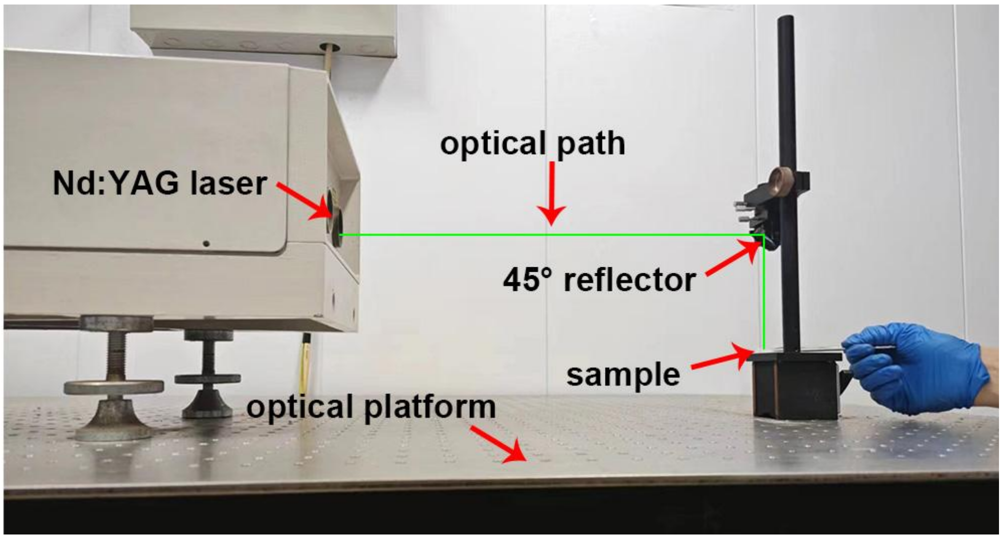

Supplementary Figure 20. The laser system used for the laser irradiation.

Supplementary Table 1. Literature studies of the fabrication of nano- and micro-sized gold spheres.

Methods	Sizes (μm)	CV	State	References
seed growth	0.005-0.15	4.8%	random	*Part Part Syst Charact* **31**, 266-273 (2014)[2]
Multi-step growth-etching	0.1-0.2	/	random	*ACS Nano* **7**, 12, 11064-11070 (2013)[3]
pulsed UV laser treatment	0.02-2	/	random	*Sci Rep* **8**, 11283 (2018)[4]
pulsed laser melting in liquid	0.05-0.25	/	random	*J Phys Chem C* **122**, 21659–21666 (2018)[5]
electroless plating	1.9	5%	random	*Adv Funct Mater* **17**, 618-622 (2017)[6]
thermal annealing	0.15-0.4	/	array	*Adv Sci* **11**, 2306239 (2024)[7]
One-pot synthesis	0.06-0.17	/	random	*Chem Mater* **33**, 7, 2593-2603 (2021)[8]
**transient emulsion self-assembly followed with laser irradiation**	**0.2-2**	**4.03%**	**array**	**This work**

Supplementary References

1. Pyatenko A, Wang H, Koshizaki N, Tsuji T. Mechanism of pulse laser interaction with colloidal nanoparticles. *Laser & Photonics Reviews* **7**, 596-604 (2013).

2. Zheng Y, Zhong X, Li Z, Xia Y. Successive, Seed-Mediated Growth for the Synthesis of Single-Crystal Gold Nanospheres with Uniform Diameters Controlled in the Range of 5–150 nm. *Part Part Syst Charact* **31**, 266-273 (2014).

3. Lee Y-J*, et al.* Ultrasmooth, Highly Spherical Monocrystalline Gold Particles for Precision Plasmonics. *ACS Nano* **7**, 11064-11070 (2013).

4. Schmidl G*, et al.* Fabrication of self-assembled spherical Gold Particles by pulsed UV Laser Treatment. *Sci Rep* **8**, 11283 (2018).

5. Tsuji T*, et al.* Stabilizer-Concentration Effects on the Size of Gold Submicrometer-Sized Spherical Particles Prepared Using Laser-Induced Agglomeration and Melting of Colloidal Nanoparticles. *J Phys Chem C* **122**, 21659-21666 (2018).

6. Li Z, Ravaine V, Ravaine S, Garrigue P, Kuhn A. Raspberry-like Gold Microspheres: Preparation and Electrochemical Characterization. *Adv Funct Mater* **17**, 618-622 (2007).

7. Chen Z*, et al.* Self-Confined Dewetting Mechanism in Wafer-Scale Patterning of Gold Nanoparticle Arrays with Strong Surface Lattice Resonance for Plasmonic Sensing. *Adv Sci* **11**, 2306239 (2024).

8. Zhang T, Li X, Li C, Cai W, Li Y. One-Pot Synthesis of Ultrasmooth, Precisely Shaped Gold Nanospheres via Surface Self-Polishing Etching and Regrowth. *Chem Mater* **33**, 2593-2603 (2021).
